# Development from *Jasminum sambac* Flower Extracts of Products with Floral Fragrance and Multiple Physiological Activities

**DOI:** 10.1155/2021/7657628

**Published:** 2021-08-12

**Authors:** Li-Chun Wu, Chieh-Li Lin, Chia-Chen Peng, Tzu-Ling Huang, Teh-Hua Tsai, Yun-Erh Kuan, Ying-Chien Chung

**Affiliations:** ^1^Department of Logistics Engineering, Dongguan Polytechnic, Dongguan 523808, China; ^2^Department of Biological Science and Technology, China University of Science and Technology, Taipei 11581, Taiwan; ^3^Department of Chemical Engineering and Biotechnology, National Taipei University of Technology, Taipei 10608, Taiwan

## Abstract

To obtain a potential commercial product with floral fragrance and physiological properties from *Jasminum sambac* flower extracts, enfleurage was conducted for a short time and followed by further extraction through supercritical fluid extraction (SFE). The product extracted through SFE (called 100%SFE) exhibited low physiological activity (including 50.7% antityrosinase activity, 38.6%–45.9% radical scavenging activity, and 6,518–15,003 mg/L half-maximal inhibitory concentration [IC_50_] of antioxidant activity) and an intense jasmine-like flavor but was nontoxic to CCD-996SK and HEMn cells. By contrast, the residue (called RO) exhibited high physiological activity (94.2%–100%), light jasmine-like flavor, and slight cytotoxicity at the concentration of 4,000 mg/L. When 100%SFE and RO were mixed in the ratio 2 : 8, the resultant mixture exhibited 100% antityrosinase activity, >91.3% radical scavenging activity, strong antioxidant activity (IC_50_: 273–421 mg/L), high total phenolic content (172.15 mg-GAE/g-extract), noncytotoxicity, and moderately intense jasmine-like flavor; it is also economically competitive. The major antioxidants in these extracts were revealed through gas chromatography-mass spectroscopy (GC-MS). Additionally, the composition and quality of fragrance were confirmed through GC-MS and sensory evaluation, respectively. The major fragrance components in the 2 : 8 extract mixture were benzyl acetate, *β*-pinene, pentadecyl-2-propyl ester, citronellol, jasminolactone, linalool, farnesol, and jasmone. On the basis of the results, we strongly suggest that the 2 : 8 mixture of extracts from *J. sambac* flowers can be a powerful antioxidant, whitening, and nontoxic ingredient that can be employed in the pharmaceutical, cosmeceutical, and food industries.

## 1. Introduction

*Jasminum sambac* Linn. (Family Oleaceae) (*J. sambac*) is an erect or scandent shrub, can grow up to roughly 1–1.5 m, and is cultivated throughout tropical and subtropical regions [1]. The flowering stage of *J. sambac* is long in Taiwan (May to October), and thus, this flower can be commercially cultivated in Taiwan for producing value-added products, such as essential oils, in both absolute and concrete forms [2]. The value-added products of *J. sambac* flowers are used extensively in the manufacture of cosmetics, perfumes, drinks, and consumer goods (e.g., toilet paper, paper tissues, and detergent) [3]. Furthermore, the flowers have been reported to have therapeutic uses in medicine for soothing irritating coughs, alleviating muscular pain [4], and treating dermatitis, diarrhea, conjunctivitis, and abdominal pain [5] as well as preventing breast cancer and stopping uterine bleeding [6]. Moreover, *J. sambac* flowers are known to have other physiological and pharmacological properties, such as antioxidant, antiaging, skin-lightening, and antibacterial activities [7], and no systemic biological toxicity or mortality was observed in tested mice [8]. The essential oils and extracts of *J. sambac* flowers are, therefore, safe as functional ingredients for general use in medicines, cosmetics, and food.

To obtain essential oils, absolute or concrete, from jasmine flowers, different extraction techniques have been employed, such as enfleurage, maceration, and solvent extraction [9]. However, poor product quality is a concern when using these methods because of solvent residues, low physiological activity, impurity, and a distorted smell [10]. Supercritical fluid extraction (SFE) is a rapid, selective, and convenient method and is usually performed with pure or modified CO_2_ [11]. It has the advantages of low operating temperature, short extraction time, favorable selectivity, high environmental compatibility, and little solvent residue [12]. Thus, SFE is considered one of the most efficient extraction methods in the food, pharmaceutical, and cosmetic industries.

Different extraction techniques, floral stages, and varieties result in different chemical constituents and concentrations in jasmine flower extracts [13]. More than 100 constituents have been identified, but linalool, benzaldehyde, benzyl alcohol, benzyl acetate, *β*-farnesene, citronellol, and nerolidol are often abundant in the extracts of *J. sambac* flowers obtained through SFE, solvent extraction, hydrodistillation, or enfleurage [1, 3, 11]. Furthermore, the variation in the chemical constituents of *J. sambac* flower extracts is dependent on the geographical origin of the *J. sambac—*that is, China, Pakistan, India, or Indonesia [7, 11, 12, 14]. The extracts have different aromas or various physiological properties, such as antioxidant, antimicrobial, antityrosinase, antitumor, and analgesic activities [15].

Commercially, an extracted product of *J. sambac* flowers should have both a floral fragrance and physiological properties. Enfleurage is used for preparing aromatic oils; however, it involves a long preparation time (e.g., 12 days) and laborious steps [3]. To solve this problem and obtain an extract with a high-grade aroma, SFE with CO_2_ is applied (SFE-CO_2_) [16]. SFE-CO_2_ is a known extraction method for low-polarity, lipophilic, or low-boiling-point volatile compounds such as aromatic compounds, but it is unfavorable for extracting compounds with polar groups (-OH, –COOH, etc.) [17]. Therefore, compounds with physiological or pharmacological activity—such as those containing polyols, polybasic acids, and polyhydroxy aromatic compounds—are found in small amounts in extracts obtained using SFE-CO_2_.

To obtain products from *J. sambac* flower extracts that have both high-grade aroma and desirable physiological properties, we prepared *J. sambac* flower extracts by using enfleurage for a short duration (6 or 24 h) and further extracted or concentrated the extracts through SFE-CO_2_. The physiological properties, cytotoxicity, and chemical constituents of the extracts, residual oils, and mixture extracts were evaluated. Finally, the floral fragrance of the four types of *J. sambac* flower mixture extracts was evaluated through a sensory test. To our knowledge, this is the first study to develop products from *J. sambac* flower extracts that have a high-grade aroma and multifunctional activities and that can be used in food, medicine, and cosmetics.

## 2. Material and Methods

### 2.1. Materials

Fresh *J. sambac* flowers were collected from agricultural fields (Huatan Township, Changhua County, Taiwan). The species were identified by Dr. Hu at the China University of Sciences and Technology (CUST), Taiwan. Voucher specimens have been deposited at the Centre for Plant Protection Studies, CUST, Taiwan. Cultures of HEMn cells (Cascade cat. C-102-5C, Cascade Biologics, Inc., Portland, OR, USA) obtained from neonatal foreskin were propagated in medium 254 supplemented with human melanocyte growth supplement (Cascade Biologics, Inc., Portland, OR, USA), and the normal human skin fibroblast cell line CCD-966SK (ATCC CRL-1881) was purchased from Bioresource Collection and Research Centre (Hsinchu, Taiwan). Mushroom tyrosinase (350 units/mL) was purchased from Sigma Chemical Co. (St. Louis, USA). All chemicals used in the current study were of analytical grade.

### 2.2. Extract Preparation

The *J. sambac* flowers were washed with distilled water to remove dirt on petals. Petals were separated from sepals, weighed, air-dried at room temperature, and then used in extraction. In the extraction, approximately 30 g of flowers was subjected to maceration with 300 mL of canola oil at room temperature for 6 or 24 h and then centrifuged at 3,000 rpm for 15 min; the products obtained were called 5% oil extract (called 5%OE) and 20% oil extract (called 20%OE), respectively. Afterward, the 20%OE product was placed into the extractor of the SFE apparatus (Separex, France), and supercritical CO_2_ extraction was performed. The extraction was conducted at 200 bar pressure and 35°C on the basis of the method detailed by Younis et al. [11]. In the separator of the SFE apparatus, CO_2_ vapor was released, leaving the extract free from CO_2_, and the extracted compounds (called 100%SFE) were collected in a flask. Furthermore, the residues in the extractor (called RO) were collected. To obtain potential commercial products (PCPs) with floral fragrance and physiological properties, 2,000 mg/L 100%SFE and 4,000 mg/L RO were mixed in various ratios: 1 : 9, 2 : 8, 3 : 7, and 4 : 6.

### 2.3. Tyrosinase Activity Measurement

The mushroom tyrosinase activity of the *J. sambac* extracts—5%OE, 20%OE, RO, 100%SFE, and PCPs—was measured through a modification of the method of Wang et al. [18]. First, 10 *μ*L of the extract was mixed with 65 *μ*L of L-tyrosine (0.03%), 105 *μ*L of phosphate-buffered saline (pH 6.8), and 20 *μ*L of tyrosinase solution in a 96-well microplate and incubated at 25 °C for 30 min in the dark. Before and after incubation, the absorbance of the reaction solution was measured at 490 nm using an Epoch ELISA reader (Bio-Tek Instruments, Winooski, VT, USA). Kojic acid and *α*-arbutin were used as positive controls. All experiments were conducted in triplicate. The antityrosinase activity of the tested sample was calculated as follows:

(1) $$\begin{array}{c} \text{antityrosinase activity }\left(\%\right)\;=\;\frac{\left[\left(A-B\right)-\left(C-D\right)\right]}{\left(A-B\right)}\times 100, \end{array}$$

where *A* is the absorbance at 490 nm without extracts (control), *B* is the absorbance at 490 nm without extracts and enzymes (blank), *C* is the absorbance at 490 nm with extracts and enzymes, and *D* is the absorbance at 490 nm without enzymes (blank of C). The half-maximal inhibitory concentration (IC_50_) of the extracts was identified as the concentration at which the tyrosinase activity was half its original value.

### 2.4. Measurement of 2,2-Diphenyl-1-Picrylhydrazyl Radical Scavenging Activity

The 2,2-diphenyl-1-picrylhydrazyl (DPPH) radical scavenging activity of the *J. sambac* extracts was measured using the method described by Wu et al. [19]. Here, 1 mL of the extract was mixed with 1 mL of ethanol (95%) and 0.5 mL of DPPH solution (100 *µ*M) and incubated at 25°C for 1 h in the dark. Before and after incubation, the absorbance of this reaction solution was measured at 517 nm by using an ultraviolet-visible (UV-vis) spectrophotometer (Shimizu, Japan). Butylated hydroxytoluene (BHT) was used as a positive control. All experiments were conducted in triplicate. The scavenging activity of the tested sample was calculated as follows:

(2) $$\begin{array}{c} \text{DPPH scavenging activity }\left(\%\right)=\left(1-\frac{A}{A_{0}}\right)\times 100, \end{array}$$

where *A*_0_ is the absorbance of the blank (without extract), and *A* is the absorbance of the test sample.

### 2.5. Measurement of 2,2′-Azino-bis(3-Ethylbenzothiazoline-6-Sulfonic Acid) Radical Scavenging Activity

The 2,2′-azino-bis(3-ethylbenzothiazoline-6-sulfonic acid) (ABTS) radical scavenging activity of the *J. sambac* extracts was measured using the method described by Lee et al. [20]. ABTS cation radical solution was prepared by mixing 7 mM ABTS with 2.45 mM K_2_S_2_O_8_ (1 : 1) for 12 h in the dark and then storing the mixture at room temperature. Before use, the ABTS solution was diluted with methanol until the absorbance at 734 nm reached 0.7. Then, 50 *μ*L of the extract was mixed with 950 *μ*L of diluted ABTS solution and incubated at 25°C for 30 min. Before and after incubation, the absorbance of this reaction solution was measured at 734 nm by using a UV-vis spectrophotometer. BHT was used as a positive control. All experiments were conducted in triplicate. The ABTS radical scavenging activity of the tested sample was calculated as follows:

(3) $$\begin{array}{c} \text{ABTS radical scavenging activity }\left(\%\right)=\left(1-\frac{A}{A_{0}}\right)\times 100, \end{array}$$

where *A*_0_ is the absorbance of the blank (without extract), and *A* is the absorbance of the test sample.

### 2.6. Measurement of Reducing Power

The ferric reducing power of the *J. sambac* extract was measured using the method described by Fejes et al. [21]. First, 1 mL of extract was mixed with 2.5 mL of phosphate buffer (0.2 M, pH 6.5) and 2.5 mL of K_3_Fe(CN)_6_ (1%); the mixture was then incubated at 50°C for 20 min. After incubation, 2.5 mL of Cl_3_CCOOH (10%) was added to terminate the reaction. After centrifugation at 4,000 *×g* for 20 min, 2.5 mL of supernatant was collected and mixed with 2.5 mL of deionized water and 0.5 mL of FeCl_3_ (0.1%) for 10 min. The absorbance was determined at 700 nm by using a UV-vis spectrophotometer. Ascorbic acid was used as a positive control. All experiments were conducted in triplicate. The IC_50_ value was defined as the effective concentration of the extract at which the absorbance was 0.5.

### 2.7. *β*-Carotene Bleaching Assay

A *β*-carotene bleaching (BCB) assay of the *J. sambac* extracts was performed using a modification of the method of Lee et al. [22]. Briefly, 20 *μ*L of *β*-carotene solution (20 mg/mL) was mixed with 1 mL of CHCl_3_, 40 *μ*L of linoleic acid, and 400 mg of Tween 40 in a round-bottomed flask, and the CHCl_3_ was then removed at 45°C in a rotary vacuum evaporator. After evaporation, 100 mL of distilled water was added, and the mixture was stirred in a sonicator. The resulting solution of volume 2.5 mL was then mixed with 350 *μ*L of extract. The solution was gently shaken and incubated at 50°C. The absorbance of the solution was measured at 470 nm by using a UV-vis spectrophotometer every 20 min until 120 min. BHT was used as a positive control. All experiments were conducted in triplicate. The antioxidant activity was calculated in terms of percentage of inhibition relative to the control by using the following equation:

(4) $$\begin{array}{c} \text{antioxidant activity }\left(\%\right)=\left(1-\frac{A}{A_{0}}\right)\times 100, \end{array}$$

where *A*_0_ is the absorbance of the blank (without extract), and A is the absorbance of the test sample. The IC_50_ values of the BCB activity of extracts were evaluated at 50% antioxidant activity.

### 2.8. Measurement of Ferrous-Ion-Chelating Activity

The ferrous-ion-chelating (FIC) activity of the *J. sambac* extracts was measured using the method described by Wu et al. [19]. First, 2 mL of extract was thoroughly mixed with 0.1 mL of FeSO_4_ (2 mM). After 30 s, 0.2 mL of ferrozine solution (5 mM) was added, and the mixture was shaken again, after which it was incubated at room temperature for 10 min. The absorbance was measured at 562 nm by using a UV-vis spectrophotometer. Ethylenediaminetetraacetic acid (EDTA) was used as a positive control. All experiments were conducted in triplicate. The FIC activity of extracts was calculated as follows:

(5) $$\begin{array}{c} \text{chelating activity }\left(\%\right)=\left(1-\frac{A}{A_{0}}\right)\times 100, \end{array}$$

where *A*_0_ is the absorbance of the blank (without extract), and *A* is the absorbance of the test sample. The IC_50_ values of the FIC activity of extracts were evaluated at 50% chelating activity.

### 2.9. Determination of Total Phenolic Content

The total phenolic content (TPC) of the various *J. sambac* extracts was estimated using a modified Folin–Ciocalteu (FC) colorimetric method and calculated in gallic acid equivalents [19]. Briefly, 1 mL of extract or gallic acid was mixed with 1 mL of the FC reagent (diluted 1/10) in a test tube and reacted at 30 °C for 10 min. Subsequently, 1 mL of 20% Na_2_CO_3_ solution was added to the mixture, which was allowed to react for 60 min in the dark. The absorbance was then measured at 725 nm by using a UV-vis spectrophotometer. The calibration curve of absorbance (*y*) versus concentration of gallic acid (*x*) was found to be *y* = 0.0501*x* + 0.0647 (*R*^2^ = 0.9968). The TPC was expressed as the gallic acid equivalent (mg-GAE/g-dried extract).

### 2.10. Determination of Total Flavonoid Content

The total flavonoid content (TFC) of the *J. sambac* extracts was estimated using a modified version of the aluminum chloride colorimetric method and calculated in catechin equivalents [23]. Briefly, 1 mL of extract or catechin was mixed with 300 *µ*L of a 5% NaNO_2_ solution and 4 mL of distilled water at 25°C for 5 min. Subsequently, 300 *µ*L of 10% AlCl_3_ solution, 2 mL of 1 M NaOH, and 2.4 mL of distilled water were added sequentially to the solution, which was then left at 25°C for 15 min. The absorbance of the solution was read at 510 nm by using a UV-vis spectrophotometer. The calibration curve of absorbance (*y*) versus concentration of catechin (*x*) was calculated as *y* = 0.0429*x* + 0.0261 (*R*^2^ = 0.9972). The TFC was expressed as the catechin equivalent (mg-CAE/g-dried extract).

### 2.11. Analysis of the Chemical Composition of the *J. sambac* Extracts

The various *J. sambac* extracts were subjected to gas chromatography-mass spectroscopy (GC-MS) (Agilent GC 7890/MS 5975, USA). To separate the volatile compounds in the extracts, 1 *μ*L of a sample volume was injected into the gas chromatograph fitted with HP-5MS capillary columns (30 *m* × 0.25 mm i.d., film thickness 0.25 *μ*m). Helium was a carrier gas with a linear velocity of 1 mL/min and a split ratio of 10 : 1. The oven temperature program ranged from 50°C to 300°C, programmed at 10°C/min, with initial and final hold times of 3 min. The interface temperature was 260°C, and the ion source temperature was 280°C. Electron impact mass spectra with ionization energy 70 eV were recorded in the 40–600 amu mass range. The chemical compositions of the extracts were identified through a comparison of the mass spectrum of the module with that of the mass spectral library from Wiley 8.0 (Wiley, New York, USA) and NITS 11 (National Institute of Standards and Technology, Gaithersburg, USA). The relative quantities of the compounds were determined through the integration of the peak area in the chromatogram.

### 2.12. Cytotoxicity Assay of the *J. sambac* Extracts

The cytotoxicity effects of the *J. sambac* extracts on CCD-966SK and HEMn cells were assessed using the 3-(4,5-dimethylthiazol-2-yl)-2,5-diphenyltetrazolium bromide (MTT) method [24]. After 24 h of incubation, the cells of density 5 × 10^6^ per wel1 (96-well plate) were washed using a fresh medium and then treated with *J. sambac* extract or the culture medium (control) for 72 h. The treated cells were then rewashed and reacted with 0.02% MTT solution at 37°C. After 4 h of MTT treatment, the medium was removed, and the precipitate in each dish was dissolved in 100 *µ*L of dimethyl sulfoxide. The absorbance of the supernatant was measured at 570 nm by using an Epoch ELISA reader. The amounts of viable cells after each treatment were expressed as the percentage of the control and calculated using the following formula:

(6) $$\begin{array}{c} \text{cell viability }\left(\%\right)=\left(\frac{A}{A_{0}}\right)\times 100, \end{array}$$

where *A*_0_ is the absorbance of the blank (without extract), and A is the absorbance of the test sample.

### 2.13. Sensory Evaluation

In total, 30 trained panelists (15 men and 15 women) were recruited to perform the sensory evaluation of PCPs derived from *J. sambac* extracts. These trained panelists were 20–22 years old, healthy, nonsmoking, and without olfactory disorders. To evaluate the fragrance emitted from the mixture extracts, 1 mL of extract was placed in a white vinyl bottle, with the bottles numbered randomly [10]. Panelists were asked to smell the samples for 1 min. The panelists assigned each PCP to one of five levels (from strongly disliked to strongly liked): very bad, bad, regular, good, and very good. The fragrance content of the PCPs was divided into five levels (0%–14%, 15%–29%, 30%–44%, 45%–59%, and 60%–74%): very low, low, acceptable, high, and very high.

## 3. Results and Discussion

### 3.1. Antityrosinase Activity

**Figure 1:**
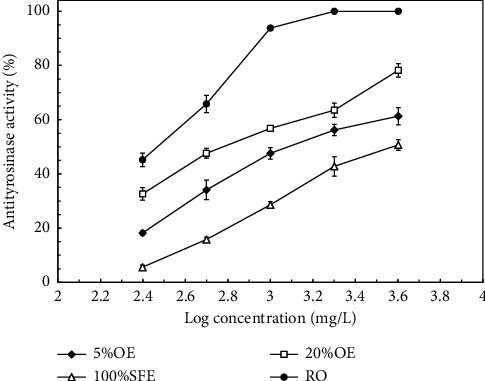
Effects of the concentrations of *J. sambac* flower extracts on mushroom tyrosinase activity. Data are expressed as the means ± standard deviations of 3 independent experiments.

Figure 1 shows the *in vitro* antityrosinase activity of the *J. sambac* flower extracts with various concentrations. The results indicated that the antityrosinase activity increased with increasing concentrations of the *J. sambac* flower extracts. The maximum antityrosinase activity increased in the following order: RO (100% ± 0.2%) > 20%OE (78.2% ± 2.5%) > 5%OE (61.3% ± 3.2%) > 100%SFE (50.7% ± 1.8%). The IC_50_ values for RO, 20%OE, 5%OE, and 100%SFE were 263.5, 618.3, 1283.7, and 3986.5 mg/L, respectively. By contrast, the IC_50_ values of the antityrosinase activity for the positive controls *a*-arbutin and kojic acid were 306.4, and 26.2 mg/L, respectively. Tyrosinase is the key enzyme for melanin formation. Excessive melanin formation in the skin may cause hyperpigmentation disorders [25]. Thus, a natural and effective tyrosinase inhibitor not only meets the demand for skin-whitening products but also lays the foundation for drugs for treating hyperpigmentation. Few studies have focused on the potential of the antityrosinase activity of *J. sambac* flower extracts. Wang et al. found that the IC_50_ of the ethanolic extract of *J. sambac* flowers reached 1,600 mg/L [25]. Thus, the extracts obtained in the present study, except 100%SFE, had potential skin-whitening properties.

### 3.2. DPPH and ABTS Radical Scavenging Activity

Antioxidants have been useful in retarding oxidative deterioration of food and cosmetics, and interest in their therapeutic potential for improving health has increased. Recent investigations have evaluated plant products' potential antioxidants against various diseases because chemical antioxidants (e.g., BHA and BHT) have been suspected of being responsible for liver damage and carcinogenesis in laboratory animals [26]. Thus, antioxidant activity is a key pharmacological property, and many pharmacological activities including skin whitening, antiaging, and anticarcinogenic activities originate from this property [27].

**Figure 2:**
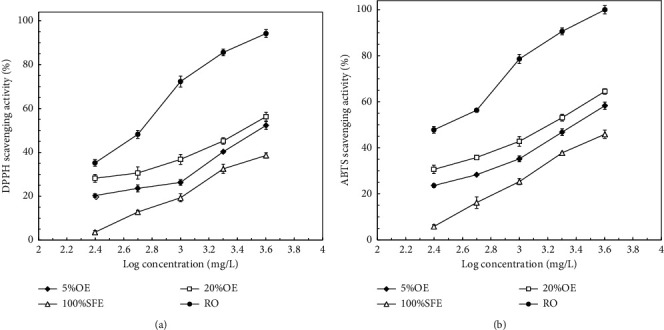
Effects of concentrations on (a) DPPH radical scavenging activity and (b) ABTS radical scavenging activity of *J. sambac* flower extracts. Data are expressed as the means ± standard deviations of 3 independent experiments.

Figure 2 shows the DPPH and ABTS radical scavenging activities of the *J. sambac* flower extracts of various concentrations. DPPH radical scavenging activity increased with increasing extract concentration (Figure 2(a)). Radical scavenging activities were significantly affected by RO concentration. The maximum DPPH radical scavenging activity increased in the following order: RO (94.2% ± 1.2%) > 20%OE (56.3% ± 1.8%) > 5%OE (52.3% ± 2.0%) > 100%SFE (38.6% ± 1.2%). The tendency was similar to that of the antityrosinase activity (Figure 1).  Figure 2(b) shows the ABTS radical scavenging activity of the *J. sambac* flower extracts with various concentrations. Results indicated that the ABTS radical scavenging activity was positively correlated with the tested concentration. When the concentration was 4,000 mg/L (log concentration: 3.6), the scavenging activity was not at its highest level. The tendency of the extracts to scavenge ABTS radicals was similar to their tendency to scavenge DPPH, but the ABTS scavenging activity was superior to that for DPPH. The IC_50_ values of DPPH and ABTS radical scavenging activities of RO were 512.6 and 368.4 mg/L, respectively; by contrast, those of the positive control BHT were 206.3 and 72.5 mg/L, respectively. The methanolic extracts of both *Jasminum grandiflorum* flowers and *J. sambac* flowers exhibited DPPH radical scavenging activity with an IC_50_ of 639 and 208 mg/L, respectively [1, 28]. The DPPH radical scavenging activity of RO originating from *J. sambac* was slightly higher than that of the methanolic extract of *J. grandiflorum* but inferior to that of the methanolic extract of *J. sambac*.

### 3.3. Reducing Power, BCB Assay, and FIC Ability

Using only a single or simple indicator to evaluate the antioxidant activity of the *J. sambac* flower extracts would not provide sufficient understanding of the extracts' antioxidant activity due to the lack of specificity and sensitivity of the methods available [29]. Therefore, reducing power, BCB activity, and FIC ability were investigated using different mechanisms to provide reliable data regarding the antioxidant capacities of the flowers.

Table 1 shows the IC_50_ values of the reducing power, BCB activity, and FIC activity for the various *J. sambac* flower extracts. A low IC_50_ indicates high activity. Different activity tendencies were observed for the three indexes. RO was the optimal solution regarding reducing power and FIC activity, but 100%SFE was the optimal solution in terms of BCB activity. In all antioxidant indexes (Figure 2 and Table 1), only the BCB activity tendency was contrary to the trend in the other antioxidant indexes. This was probably because the *ß*-carotene solution is lipophilic, which makes it favor hydrophobic 100%SFE products to function. The IC_50_ values of the reducing power, BCB activity, and FIC activity for RO and 100%SFE were 251.5 ± 23.5, 623.8 ± 21.3, and 382.0 ± 18.5 mg/L and 6,518.2 ± 351, 125.6 ± 5.8, and 15,003 ± 201 mg/L, respectively. By contrast, the IC_50_ values of the reducing power, BCB activity, and FIC activity for the positive controls ascorbic acid, BHT, and EDTA were 61.3, 92.5, and 20.5 mg/L, respectively. Compared with the IC_50_ of the reducing power for the methanolic extract of *J. grandiflorum* (603.66 mg/L), RO derived from *J. sambac* flower extracts was promising [28]. These results indicate that when our extraction process is employed, *J. sambac* flower extracts can not only scavenge free radicals (Figure 2) but also efficiently inhibit chain reactions (Table 1).

### 3.4. TPC and TFC

Phenolic substances exhibit health-promoting activities, including antipathogenic, antiradiation, antitumor, and antioxidant activities [30]. Polyphenols can be classified into four categories: flavonoids, phenolic acids, stilbene, and lignans [31]. Therefore, TPC and TFC should be simultaneously evaluated to understand the antioxidant characteristics of natural plants.

Table 2 lists the TPC and TFC of the various *J. sambac* flower extracts. The TPC and TFC ranged from 20.65 ± 0.82 to 182.36 ± 9.23 mg-GAE/g-extract and 4.65 ± 0.32 to 10.35 ± 1.02 mg-CAE/g-extract, respectively. RO had the highest TPC and TFC, whereas 100%SFE had the lowest. The TPC and TFC of the *J. sambac* flower extracts were clearly higher than or equivalent to those of *J. sambac* flower extracts obtained using acetone/water/acetic acid solvent (20.64 mg-GAE/g-extract and 4.44 mg-CAE/g-extract, respectively) [30]. Studies have reported positive correlations between antioxidant capacities and TPC as well as TFC [32]. In this study, the antioxidant capacity of the *J. sambac* flower extracts was found to be positively related to their TPC and TFC. That is, the more TPC and TFC were, the higher the antioxidant capacity was.

### 3.5. Cytotoxic Effects

**Figure 3:**
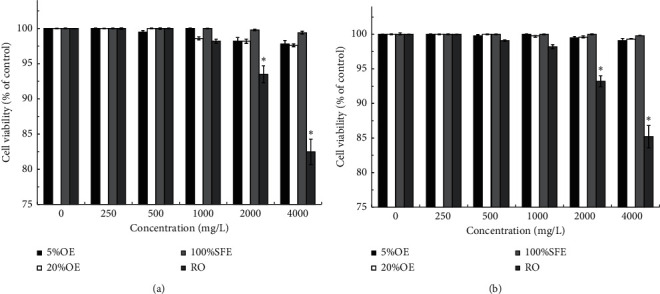
Cell viability of (a) CCD-966SK cells and (b) HEMn cells treated by *J. sambac* flower extracts with various concentrations for 72 h. Data are expressed as the means ± standard deviations of 3 independent experiments ( ^*∗*^*P* < 0.05 versus blank control).

The MTT assay is a common method of evaluating the cytotoxicity of chemical compounds by observing cell viability [33]. Although some studies in the field of cosmetics have evaluated antityrosinase, collagenase, elastase, and hyaluronidase activities and obtained IC_50_ values of 250–1,600 mg/L [7,12], cytotoxicity evaluations have not been performed. To ensure user safety, the cytotoxic effects of *J. sambac* flower extracts on human cells should be assessed.  Figure 3 shows the effects of the *J. sambac* flower extracts with different concentrations on the growth of human CCD-966SK and HEMn cells after 72 h of incubation. At low extract concentrations (0–1,000 mg/L), cell viability exceeded 98.2% and cytotoxicity was nonsignificant compared with the control (*P* > 0.05) for all extracts. When the RO concentration was 2,000 mg/L, the viability of CCD-966SK and HEMn cells was slightly lower at 93.5% ± 1.2% and 93.2% ± 0.8%, respectively. When the RO concentration was increased to 4,000 mg/L, clear effects were observed and the viability of CCD-966SK and HEMn cells was decreased to 82.5% ± 1.8% and 85.2% ± 1.6%, respectively. Although cell viability was affected by RO concentrations 2,000–4,000 mg/L compared with the control, RO was not toxic to the cells because cell viability remained ≥80% [34]. The relatively high cytotoxicity of RO may be related to its high physiological activity (Figures 1 and 2 and Table 1). Thus, *J. sambac* flower extracts are safe for possible applications in the pharmaceutical, health food, and cosmetic industries.

### 3.6. Effects of the Extract Mixture Ratio on Antityrosinase Activity, Radical Scavenging Activity, and Cell Viability

**Figure 4:**
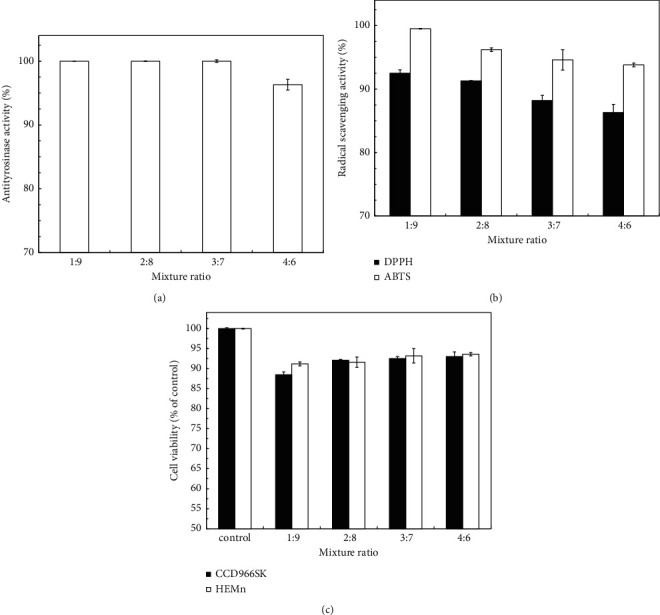
Effects of mixture ratio of *J. sambac* flower extracts on (a) antityrosinase activity, (b) radical scavenging activity, and (c) cell viability. The potential commercial product was produced by mixing 2,000 mg/L 100%SFE and 4,000 mg/L RO at various ratios. Data are expressed as the means ± standard deviations of 3 independent experiments.

On the basis of the physiological activity and cytotoxicity results for a single *J. sambac* flower extract and considering floral fragrance and the cost of a single *J. sambac* flower extract, PCPs were produced by mixing 2,000 mg/L 100%SFE and 4,000 mg/L RO in various ratios. 100%SFE was found not only to have relatively low physiological activity and to be noncytotoxic but also to have the most fragrance and be the most expensive. On the contrary, RO had high physiological activity and relatively high cytotoxicity but the weak scent and was the cheapest. In the SFE procedure, RO is often discarded; hence, the cost of 100%SFE is approximately 10–20 times that of RO.  Figure 4 shows the effects of mixture ratio on antityrosinase activity, radical scavenging activity, and cell viability. The effects of 100%SFE and RO in various ratios on antityrosinase activity were nonsignificant (*P* > 0.05) except that at the 4 : 6 mixture ratio, the antityrosinase activity was slightly lower at 96.3% ± 0.84%.

The DPPH and ABTS radical scavenging activities decreased with a decrease in RO concentration (i.e., an increase in 100%SFE concentration; Figure 4(b)). The mixtures had higher scavenging activity for ABTS free radicals than DPPH free radicals. However, little difference was discovered in the DPPH and ABTS scavenging activities between the different extract mixtures; the maximum difference was approximately 6.2%.  Table 3 lists the antioxidant activity (reducing power, BCB activity, and FIC activity) and antioxidant capacity (TPC and TFC) of the *J. sambac* flower extracts at various mixture ratios. The results revealed that the 1 : 9 mixture ratio (100%SFE: RO; V/V) had the lowest IC_50_ for reducing power and FIC activity but the highest IC_50_ in the BCB assay. Both TPC and TFC decreased with an increase in the 100%SFE content, indicating that 100%SFE contained fewer antioxidants. Even so, the TPC and TFC of all the mixtures were significantly greater (1.7–6.7 times) than those of *J. sambac* flower extracts obtained using acetone/water/acetic acid solvent [30].

Figure 4(c) shows the effects of the mixture ratio on cell viability. When 100%SFE and RO were mixed in volume ratios 1 : 9, 2 : 8, 3 : 7, and 4 : 6, the 100%SFE and RO concentrations (mg/L) of the mixture solution were equivalent to 200 and 3,600; 400 and 3,200; 600 and 2,800; and 800 and 2,400, respectively. The results showed that the viability of CCD-966SK cells (88.5% ± 0.7% to 93.5% ± 1.2%) and HEMn cells (91.2% ± 0.5% to 93.6% ± 0.4%) was similar for all PCPs.  Table 3 indicates that the IC_50_ for the reducing power, BCB activity, and FIC activity of various mixture extracts was 258 ± 18.6 to 324 ± 12.8, 456 ± 34.1 to 602 ± 36.4, and 394 ± 32.1 to 512 ± 46.2 mg/L, respectively. At these concentration ranges, the PCPs do not inhibit cell viability, even if 100% physiological activity is achieved. Thus, the PCPs are safe for commercial application.

### 3.7. Chemical Compositions of *J. sambac* Flower Extracts and the 2 : 8 Extract Mixture

To understand the physiological composition and aroma components, various *J. sambac* flower extracts were analyzed through GC-MS.  Table 4 lists the detectable chemical components of the *J. sambac* flower extracts and 2 : 8 extract mixture. The results indicated that the number of chemical components in 5%OE, 20%OE, 100%SFE, RO, and the 2 : 8 extract mixture was 45, 39, 29, 38, and 42, respectively. These components generally belonged to three classes, namely, phenylpropanoid/benzenoid, terpenoid, and fatty acid esters; the result was in agreement with that of a previous report [10]. 5%OE and 100%SFE had the most and least chemical components, respectively. After the extraction process, the number of chemical components was lower. The main components (>2%) of the products (5%OE and 20%OE) of *J. sambac* flower extracts obtained using an oil solvent were benzyl acetate, *β*-pinene, hexadecan-1-ol, 9,12,15-octadecatrienoic acid, citronellol, octacosane, squalene, floridanine, and jasminolactone. Among them, the content of jasminolactone (17.14%–19.29%), floridanine (9.96%–10.59%), and citronellol (6.10%–8.94%) was relatively high. Furthermore, a similar composition was found when *J. sambac* flowers were extracted through enfleurage [3].

The main components (>2%) of 100%SFE were benzyl acetate (6.18%), *β*-pinene (5.03%), pentadecyl-2-propyl ester (2.06%), 9,12,15-octadecatrienoic acid (4.37%), hexadecan-1-ol (2.99%), nerolidol (2.20%), citronellol (8.47%), floridanine (22.52%), and jasminolactone (16.91%). The amounts of hydrophobic or volatile fragrance components in 100%SFE—such as linalool, 2-phenyl ethyl acetate, geranyl acetate, *β*-caryophyllene, farnesol, jasmone, and pentadecyl-2-propyl ester—were high compared with those in 20%OE, indicating the nature of the SFE-CO_2_ technique [17]. Among them, benzyl acetate produces a strong jasmine aroma, and other flavors such as citronellol, farnesol, linalool, and jasmone are famous fragrance ingredients used in perfumes, cosmetics, and household products [35,36]. Moreover, citronellol, farnesol, and geranyl acetate were detected in Pakistan *J. sambac* flower extracts obtained using the SFE-CO_2_ technique [11]. Furthermore, benzyl acetate, linalool, and *ß*-caryophyllene were present in China *J. sambac* flower extracts obtained using the SFE–diether methyl ester technique [12]. Linalool, benzyl acetate, and *ß*-caryophyllene were found in China *J. sambac* flower extracts obtained using the SFE-petroleum ether technique [10].

After the SFE procedure, the types of chemical components in RO were similar to those in 20%OE. This was reasonable because both 100%SFE and RO were derived from 20%OE. Partial antioxidant ingredients were found only in RO but not in 100%SFE; they included 3,7,11,15-tetramethyl-1-hexadecen-3-ol, 3,7,11-trimethyl-1,6,10-dodecatrien-3-ol, *α*-cadinol, 3,7,11,15-tetramethyl-1-hexadecen-3-ol, benzyl alcohol, eugenol, *β*-sitosterol, jasminol, linalyl-*β*-D-glucopyranoside, and 1-hentriacontanol. Thus, the antioxidant activity of RO was much higher than that of 100%SFE. The relevant results are presented in Figures 1 and 2 and Tables 1 and 2. The number of different chemical components in the 2 : 8 extract mixture was higher than that in the pure *J. sambac* flower extracts except for 5%OE. The ingredients in the 2 : 8 extract mixture contained both antioxidants with a hydroxyl functional group and volatile fragrance components, similar to the ingredients of natural *J. sambac* flower extracts [2,5]. Additionally, palmitic acid, linoleic acid, and oleic acid are almost never found in *J. sambac* flower extracts, and they may originate from the extracted solvent—that is, mustard oil [37]. The types and amounts of antioxidant constituents in the RO and 100%SFE appropriately explained and reflected the antioxidant activity findings presented in previous sections.

### 3.8. Sensory Evaluation

Because the flavor and fragrance of commercial products are critical, sensory evaluation is always required [38].  Table 5 lists the results of the sensory and fragrance quality evaluations for the *J. sambac* flower extracts at different mixture ratios (100%SFE to RO) analyzed through GC-MS. The results clearly indicated that the higher the proportion of 100%SFE within the solution, the stronger the fragrance. This may have been due to the high volatility of the flavor content in the extract mixture [3]. The 2 : 8 extract mixture exhibited a moderately intense, fresh, and jasmine-like flavor. Regarding the content percentage of fragrant compounds in the various extract mixtures, the content of the 2 : 8 extract mixture was classified as acceptable (42.6%), whereas that of the 4 : 6 mixture was classified as high (51.3%) due to the accumulation of fragrance components. Furthermore, the 2 : 8 and 4 : 6 extract mixtures contained 18 and 26 flavor components, respectively. Considering overall physiological activity, cytotoxicity, flavor acceptability, and product cost, the 2 : 8 jasmine extract mixture was identified as the optimal commercial product.

## 4. Conclusion

Our study is the first to provide detailed characteristics of commercial products obtained from *J. sambac* flower extract mixtures with a combination of fragrance and multifunctional physiological activities. RO was mostly composed of antioxidant ingredients with strong antityrosinase activity, high antioxidant activity, and slight cytotoxicity. By contrast, 100%SFE was composed of few antioxidant ingredients but abundant flavor compounds (e.g., benzyl acetate, citronellol, farnesol, linalool, and jasmone) compared with other *J. sambac* flower extracts, resulting in relatively low physiological activities, strong aroma, and noncytotoxicity. Considering the advantages and disadvantages of both, 100%SFE and RO were mixed in the ratio 2 : 8, and the resultant mixture exhibited satisfactory multifunctional physiological activities, noncytotoxicity, and moderately intense jasmine-like flavor and was economical. Similar products have not been reported in the literature or exist in the market. Thus, we strongly suggest that the 2 : 8 extract mixture derived from *J. sambac* flowers is a powerful antioxidant and whitening ingredient that can be employed in the food, cosmeceutical, and even pharmaceutical industries.

## Figures and Tables

**Table 1:** Antioxidant activity (reducing power, *β*-carotene bleaching assay, and ferrous-chelating activity) of *J. sambac* flower extracts.

	5%OE	20%OE	100%SFE	RO
Reducing power, IC_50_ (mg/L)	2,017 ± 135.2	852 ± 58.1	6,518.2 ± 351	251.5 ± 23.5
*β*-Carotene bleaching assay, IC_50_ (mg/L)	356 ± 35.1	256 ± 18.2	125.6 ± 5.8	623.8 ± 21.3
Fe(II) chelating activity, IC_50_ (mg/L)	10,253 ± 358	1,823 ± 105.2	15,003 ± 201	382.0 ± 18.5

**Table 2:** Total phenolic content (TPC) and total flavonoid content (TFC) of *J. sambac* flower extracts.

	5%OE	20%OE	100%SFE	RO
TPC (mg-GAE/g)	31.21 ± 1.81	43.65 ± 2.26	20.65 ± 0.82	182.36 ± 9.23
TFC (mg-CAE/g)	6.32 ± 0.18	8.14 ± 0.45	4.65 ± 0.32	10.35 ± 1.02

**Table 3:** Antioxidant activity (reducing power, *β*-carotene bleaching assay, and ferrous-chelating activity) and antioxidant capacity (TPC and TFC) of *J. sambac* flower extracts at various mixture ratios.

	1 : 9^*∗*^	2 : 8	3 : 7	4 : 6
Reducing power, IC_50_ (mg/L)	258 ± 18.6	273 ± 24.2	306 ± 30.1	324 ± 12.8
*β*-Carotene bleaching assay, IC_50_ (mg/L)	602 ± 36.4	547 ± 20.8	512 ± 16.5	456 ± 34.1
Fe(II) chelating activity, IC_50_ (mg/L)	394 ± 32.1	421 ± 38.5	466 ± 32.8	512 ± 46.2
TPC (mg-gae/g)	180.43 ± 10.36	172.15 ± 8.75	163.89 ± 12.74	138.95 ± 7.87
TFC (mg-cae/g)	10.02 ± 0.92	9.17 ± 1.24	8.35 ± 0.63	7.54 ± 0.83

^*∗*^The PCP was produced by mixing 2,000 mg/L 100%SFE and 4,000 mg/L RO at various mixture ratios.

**Table 4:** Chemical composition of *J. sambac* flower extracts and the 2 : 8 mixture.

Retention time (min)	Chemical compounds	5%OE	20%OE	100%SFE	RO	Mixture
7.935	Benzyl acetate	3.08	3.2	6.18	4.18	3.78
8.240	Limonene	1.5	1.14	1.12	1.89	1.32
24.056	Methyl salicylate	0.69	0.58	—	—	0.54
24.158	n-Dodecane	1.43	1.36	1.58	0.66	1.23
24.482	3,7,11,15-Tetramethyl-1-Hexadecen-3-ol	0.96	—	—	0.8	—
24.819	3,7,11-Trimethyl-1,6,10-dodecatrien-3-ol	0.89	—	—	0.81	0.66
24.934	Germacrene D	1.1	0.84	1.10	0.90	0.85
25.106	Benzyl alcohol	0.6	—	—	0.68	—
26.340	Linalool	0.79	—	1.12	—	0.71
26.925	Decane	0.67	0.81	1.03	—	0.68
27.008	*α-*Cadinol	1.07	1.05	—	0.81	0.88
27.167	*β-*Elemene	1.48	1.54	—	—	1.26
27.205	2-Phenyl ethyl acetate	0.84	0.84	1.27	—	0.76
27.313	*β-*Pinene	3.11	4.63	5.03	1.89	3.09
27.409	Geraniol	0.95	1.00	1.20	0.63	0.84
27.676	Geranyl acetate	1.41	1.18	1.48	0.87	1.11
27.784	*β-*Caryophyllene	1.39	0.85	1.02	0.85	0.79
27.841	Pentadecyl-2-propyl ester	1.45	1.66	2.06	0.75	1.42
28.013	Farnesol	1.87	1.23	1.51	1.67	1.18
28.115	Eugenol	0.87	—	—	0.95	—
28.280	*β-*Sitosterol	0.84	—	—	0.95	0.68
28.414	Palmitic acid	0.93	0.67	—	0.86	0.71
28.605	Hexadecan-1-ol	2.61	3.38	2.99	1.59	2.96
29.018	9,12,15-Octadecatrienoic acid methyl ester	1.15	1.58	1.73	1.00	1.31
29.565	Linoleic acid	—	1.15	1.15	0.64	—
29.623	Phytol	1.03	1.46	1.44	0.96	1.21
29.718	Tricosane	0.87	—	—	1.16	0.95
29.724	Octadecanoic acid methyl ester	—	1.21	1.12	—	—
29.782	Oleic acid	1.06	1.42	—	—	0.89
30.017	3,7,11,15-Tetramethyl-1-hexadecen-3-ol	0.76	1.38	—	0.75	0.62
30.138	9,12,15-Octadecatrienoic acid	2.82	4.10	4.37	2.84	2.97
30.857	Nerolidol	1.98	1.84	2.20	—	1.64
31.448	Pentadecane	1.07	0.72	—	1.86	0.93
31.913	Citronellol	6.10	8.94	8.47	5.37	7.85
33.764	Heptadecane	1.20	1.32	1.89	1.98	1.33
33.974	Octacosane	2.17	2.13	1.00	—	1.15
36.926	Jasmone	1.46	1.2	1.46	2.64	1.42
40.184	Nonadecane	1.06	0.99	1.57	2.01	1.47
42.111	*α-*Farnesene	1.56	0.90	1.31	2.04	1.31
43.536	Linalyl *β-*D-glucopyranoside	0.9	0.76	—	1.51	0.81
44.147	Jasminol	0.75	—	—	1.17	0.71
44.236	Tetratetracontane	0.56	0.74	—	1.31	0.88
44.707	1-Hentriacontanol	0.53	0.45	—	2.15	1.14
46.355	Eicosane	0.78	0.89	—	0.66	1.17
46.876	Squalene	7.61	1.15	1.11	11.07	1.98
52.640	Floridanine	9.96	10.59	22.52	13.86	17.63
55.630	Jasminolactone	17.14	19.29	16.91	16.35	15.53

**Table 5:** Sensory evaluation and fragrance quality of *J. sambac* flower extracts at different mixture ratios analyzed through GC-MS.

	1 : 9	2 : 8	3 : 7	4 : 6
Sensory evaluation of fragrance	Regular	Good	Very good	Very good
Levels of fragrance analyzed by GC-MS	Low	Acceptable	Acceptable	High
Number of major fragrance composition by GC-MS	9	18	21	26

## Data Availability

The datasets generated and analyzed during the current study are available from the corresponding author upon reasonable request.
